# Will the age of peak ultra-marathon performance increase with increasing race duration?

**DOI:** 10.1186/2052-1847-6-36

**Published:** 2014-09-27

**Authors:** Christoph Alexander Rüst, Matthias Alexander Zingg, Thomas Rosemann, Beat Knechtle

**Affiliations:** 1 Institute of Primary Care, University of Zurich, Zurich, Switzerland; 2 Gesundheitszentrum St. Gallen, St. Gallen, Switzerland; 3 Facharzt FMH für Allgemeinmedizin, Gesundheitszentrum St. Gallen, Vadianstrasse 26, 9001 St. Gallen, Switzerland

**Keywords:** Master athlete, Female, Male, Ultra-endurance

## Abstract

**Background:**

Recent studies found that the athlete’s age of the best ultra-marathon performance was higher than the athlete’s age of the best marathon performance and it seemed that the athlete’s age of peak ultra-marathon performance increased in distance-limited races with rising distance.

**Methods:**

We investigated the athlete’s age of peak ultra-marathon performance in the fastest finishers in time-limited ultra-marathons from 6 hrs to 10 d. Running performance and athlete’s age of the fastest women and men competing in 6 hrs, 12 hrs, 24 hrs, 48 hrs, 72 hrs, 144 hrs (6 d) and 240 hrs (10 d) were analysed for races held between 1975 and 2012 using analysis of variance and multi-level regression analysis.

**Results:**

The athlete’s ages of the ten fastest women ever in 6 hrs, 12 hrs, 24 hrs, 48 hrs, 72 hrs, 6 d and 10 d were 41 ± 9, 41 ± 6, 42 ± 5, 46 ± 5, 44 ± 6, 42 ± 4, and 37 ± 4 yrs, respectively. The athlete’s age of the ten fastest women was different between 48 hrs and 10 d. For men, the athlete’s ages were 35 ± 6, 37 ± 9, 39 ± 8, 44 ± 7, 48 ± 3, 48 ± 8 and 48 ± 6 yrs, respectively. The athlete’s age of the ten fastest men in 6 hrs and 12 hrs was lower than the athlete’s age of the ten fastest men in 72 hrs, 6 d and 10 d, respectively.

**Conclusion:**

The athlete’s age of peak ultra-marathon performance did not increase with rising race duration in the best ultra-marathoners. For the fastest women ever in time-limited races, the athlete’s age was lowest in 10 d (~37 yrs) and highest in 48 hrs (~46 yrs). For men, the athlete’s age of the fastest ever in 6 hrs (~35 yrs) and 12 hrs (~37 yrs) was lower than the athlete’s age of the ten fastest in 72 hrs (~48 yrs), 6 d (~48 yrs) and 10 d (~48 yrs). The differences in the athlete’s age of peak performance between female and male ultra-marathoners for the different race durations need further investigations.

## Background

Ultra-marathon running is of high popularity [1-3] where an ultra-marathon is defined as any running distance longer than the classical marathon of the 42.195 km distance [4]. Most frequently, ultra-marathon races are held in distance-limited races in km from 50 km to 100 km, in distance-limited races in miles from 50 miles to 100 miles and in time-limited races from 6 hrs to 24 hrs [4].

Recent studies reported the athlete’s age of the best marathon [5] and the best ultra-marathon [3,6] performance. For marathoners, Hunter *et al.*[5] reported that the fastest marathoners competing in the ‘World Marathon Majors Series’ in the last 30 years were at the age of 29.8 ± 4.2 yrs in women and 28.9 ± 3.8 yrs in men. For ultra-marathoners competing in distance-limited races, it seemed that the athlete’s age of peak ultra-marathon performance was higher compared to marathoners [5] and it seemed to increase with rising race distance [3,6]. For the annual ten fastest women and men competing in all 100-km ultra-marathons held worldwide between 1960 and 2012, the athlete’s age of peak ultra-marathon performance remained unchanged at 34.9 ± 3.2 and 34.5 ± 2.5 yrs, respectively [6]. In 161-km ultra-marathoners, however, the age of the fastest athletes was higher [3] compared to the age of the fastest 100-km ultra-marathoners [6]. The mean athlete’s age of the annual top ten women and men competing between 1998 and 2011 in 161-km ultra-marathons was 39.2 ± 6.2 and 37.2 ± 6.1 yrs, respectively [3]. The athlete’s age of peak ultra-marathon performance was not different between women and men and showed no changes across years of competition [3].

For ultra-marathoners, the athlete’s age of peak ultra-marathon performance has been investigated for single races [7,8] or single distances [3,9,10]. However, the assumption that the athlete’s age of peak ultra-marathon performance will increase with rising distance or ascending duration of a race needs verification. A very recent study investigating distance-limited ultra-marathons from 50 to 3,100 miles found for men that the age of peak running speed increased with increasing race distance to ~45 yrs in 1,000 miles, whereas it decreased to ~39 yrs in 3,100 miles. In women, the upper age of peak running speed increased to ~51 years in 3,100-miles [11]. However, this study lacked the direct comparison between the ages of the fastest runners for each distance and the conclusion that runners in their forties dominate ultra-marathons from 50 to 3,100 miles was based upon approximate comparisons but not statistical analyses [11].

Apart from distance-limited races, also time-limited races do exist [12]. Distance- and time-limited ultra-marathons are basically different since athletes in a distance-limited race have to finish within the time limit whereas athletes in a time-limited race have to cover as many km as possible within a defined time [12]. However, there is a difference in the number of the most often offered time- and distance-limited races. While there are eight different race durations in the time-limited races (*i.e.* 6 hrs, 12 hrs, 24 hrs, 48 hrs, 72 hrs, 6 d and 10 d, there are nine different race distances in the distance-limited races (*i.e.* 50 km, 100 km, 200 km, 1,000 km, 50 miles, 100 miles, 200 miles, 1,000 miles and 3,100 miles).

Therefore, the assumption that the age of peak ultra-marathon performance increases with increasing race distance [11] must be verified. The first aim of the study was to determine the athlete’s age of peak ultra-marathon performance in time-limited races from 6 hrs to 10 d during the 1975–2012 period and it was hypothesized that the athlete’s age of peak ultra-marathon performance would increase with rising duration of the events. Since most of the athletes competing in time-limited ultra-marathons were starting in the shorter distances (*i.e.* 50 and 100 miles) and only a small number of athletes competed in longer distances (*i.e.* 200 to 3,100 miles) [11], we also analysed the participation trends for the time-limited races.

## Methods

### Ethics

The study was approved by the Institutional Review Board of St. Gallen, Switzerland, with a waiver of the requirement for informed consent given that the study involved the analysis of publicly available data.

### Data sampling and data analysis

Data were retrieved from the website of Deutsche Ultramarathon Vereinigung (DUV) http://www.ultra-marathon.org[13]. This website records in the section statistics [14] all race results of ultra-marathons held worldwide. Race results of ultra-marathons held between 1975 and 2012 in time-limited races such 6 hrs, 12 hrs, 24 hrs, 48 hrs, 72 hrs, 144 hrs (6 d) and 240 hrs (10 d) were collected. Performances of the annual top and the annual top ten women and men were determined. Additionally, the performances of the ten fastest women and men ever for each race were determined. To allow the comparability between the different race times of 6 hrs and 10 d, all achieved distances (km) were converted to running speed (km/h) using the equation [running speed (km/h)] = [race distance (km)]/[race time (h)]. If less than ten athletes were available in a certain year for a certain race time, that year and all race times was excluded from analysis.

### Statistical analysis

Each set of data was tested for normal distribution and for homogeneity of variances prior to statistical analyses. Normal distribution was tested using a D’Agostino and Pearson omnibus normality test and homogeneity of variances was tested using a Levene’s test. Trends in participation were analysed using regression analysis with ‘straight line’ and ‘exponential growth equation’ model, whereas for each set of data (*e.g.* each sex) both models where compared using Akaike’s Information Criteria (AICc) to decide which model showed the highest probability of correctness. Differences in performance and athlete’s age of peak performance between race durations were investigated using one-way analysis of variance (ANOVA) with subsequent Tukey-Kramer post-hoc analysis. To investigate changes in performance and in athlete’s age across years of competition, single and multi-level regression analyses were used. A hierarchical regression model was used to avoid the impact of a cluster-effect on results in case one athlete finished more than once in the annual top or top ten. Regression analyses of performance were corrected for the age of the athletes to prevent a misinterpretation of an ‘age-effect’ as a ‘time-effect’. Statistical analyses were performed using IBM SPSS Statistics (Version 21, IBM SPSS, Chicago, IL, USA) and GraphPad Prism (Version 6.01, GraphPad Software, La Jolla, CA, USA). Significance was accepted at *p* < 0.05 (two-sided for *t*-tests). Data in the text are given as mean ± standard deviation (SD).

## Results

Data from 86,451 runners (*i.e.* 17,901 women and 68,550 men) were available. A total of 1,502 women and 5,099 men had to be excluded from data analysis due to missing information on age in the race results. Overall, data from 16,399 women and 63,451 men were complete and included in the data analysis.

### Participation trends

**Figure 1:**
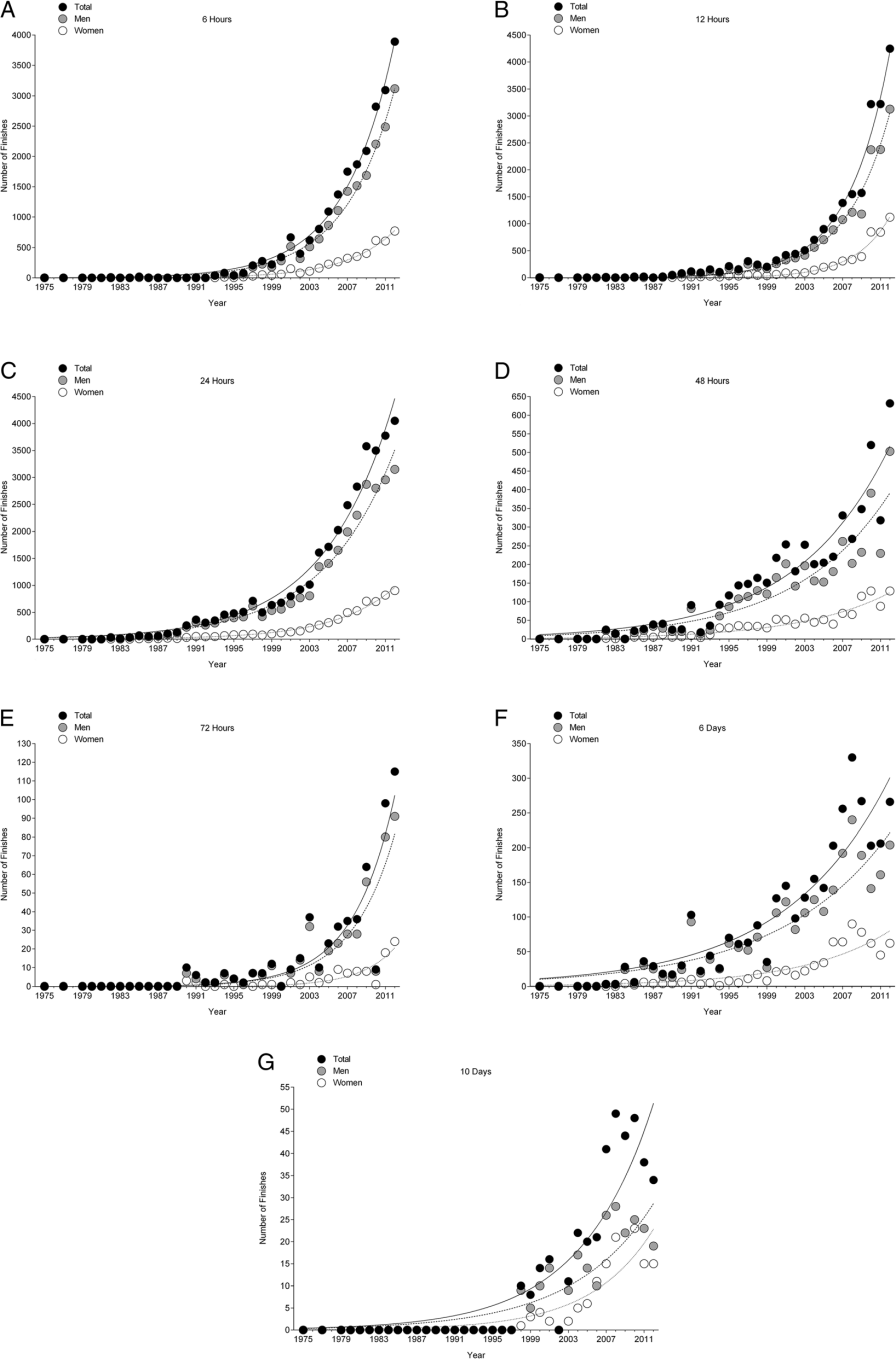
Changes in the numbers of finishes across the years in 6 hrs (Panel A), 12 hrs (Panel B), 24 hrs (Panel C), 48 hrs (Panel D), 72 hrs (Panel E), 6 d (Panel F) and 10 d (Panel G).

**Figure 2:**
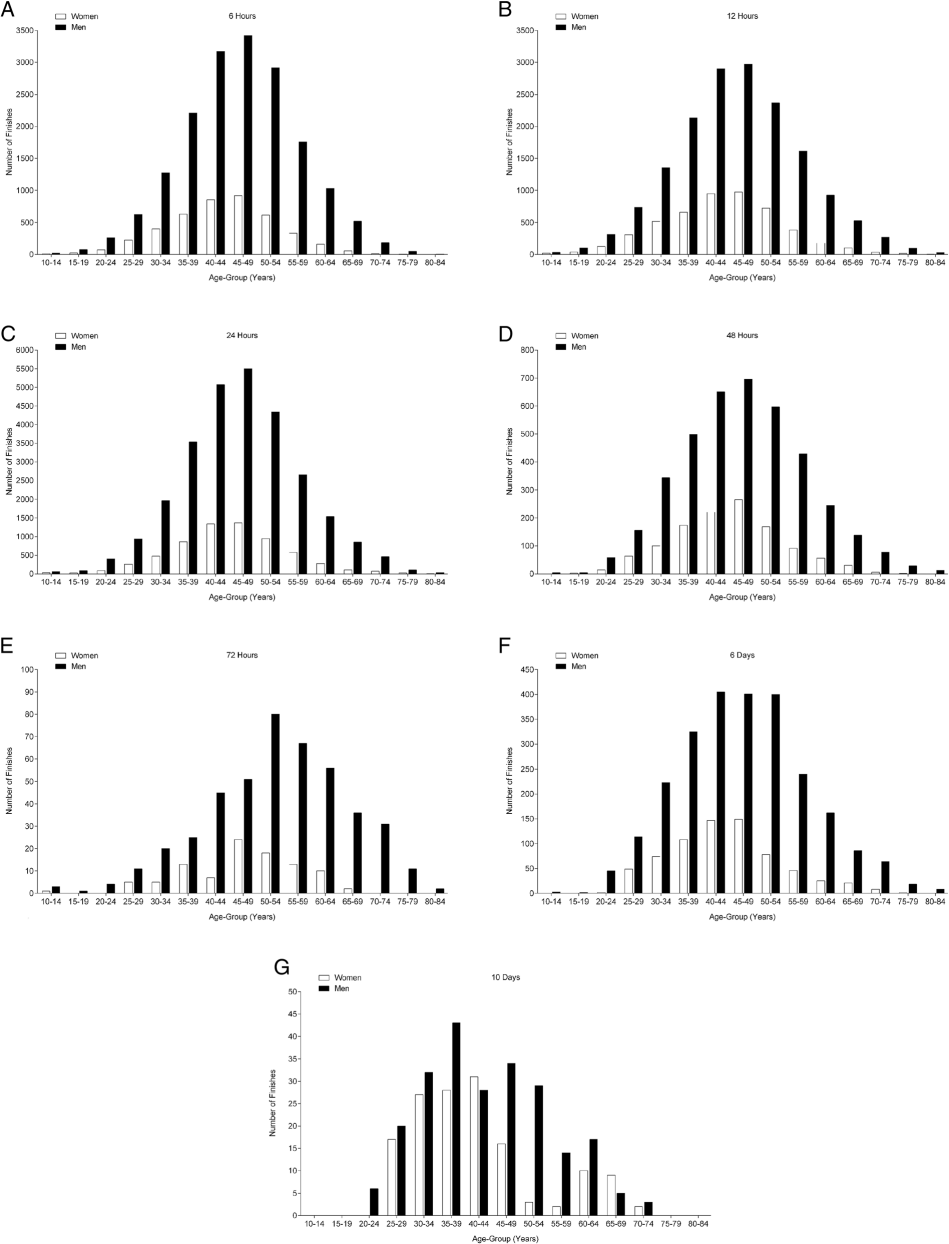
Total finishes in age groups in 6 hrs (Panel A), 12 hrs (Panel B), 24 hrs (Panel C), 48 hrs (Panel D), 72 hrs (Panel E), 6 d (Panel F) and 10 d (Panel G).

For all events from 6 hrs to 10 d, an exponential increase in participation was found in both women and men (Figure 1). The number of all events held was highest in 12 hrs but most of the finishes were recorded in 24 hrs for both women and men. In 6 hrs to 48 hrs, the highest number of athletes was recorded in age group 45–49 yrs for both women and men (Figure 2). For longer race durations, the distribution was different. In 72 hrs, the highest number of women was recorded in age group 45–49 yrs and the highest number of men in age group 50–54 yrs. In 6 d, the highest number of athletes was in age group 45–49 yrs for women and 40–44 yrs for men. For 10 d, the number of recorded athletes was highest in the age group 40–44 yrs for women and 35–39 yrs for men.

### Age and running speed of the ten fastest ever

**Figure 3:**
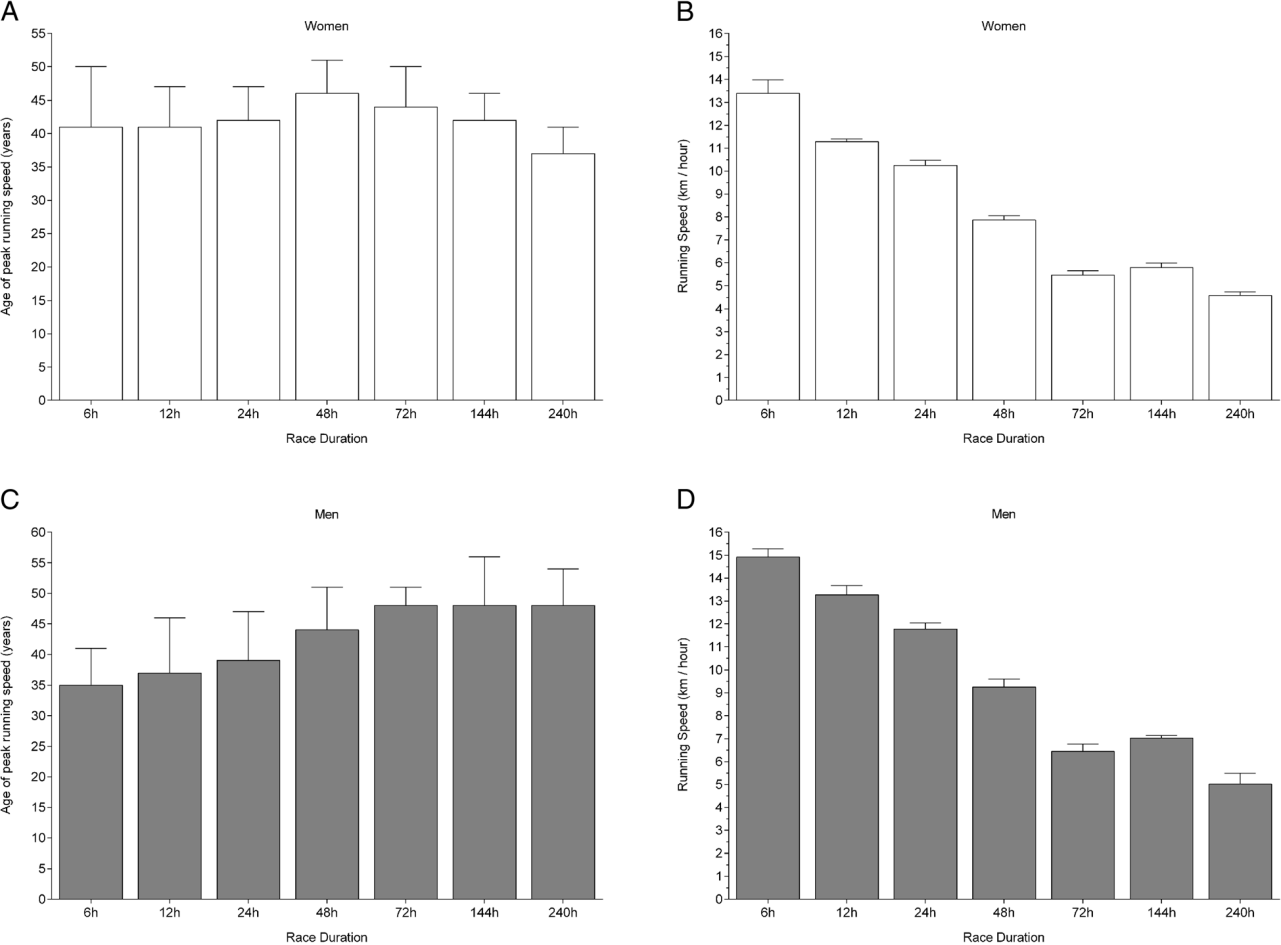
**Differences in performance and age of peak performance between race durations for the ten fastest women and men ever.** Age **(Panel A)** and running speed for women **(Panel B)** and age **(Panel C)** and running speed **(Panel D)** for men.

The athlete’s age of the ten fastest women ever was 41 ± 9 yrs (6 hrs), 41 ± 6 yrs (12 hrs), 42 ± 5 yrs (24 hrs), 46 ± 5 yrs (48 hrs), 44 ± 6 yrs (72 hrs), 42 ± 4 yrs (6 d), and 37 ± 4 yrs (10 d) (Figure 3). The athlete’s age of the ten fastest women was different between 48 hrs and 240 hrs, no differences were found between the other race durations (Table 1). For men, the athlete’s ages were 35 ± 6 yrs (6 hrs), 37 ± 9 yrs (12 hrs), 39 ± 8 yrs (24 hrs), 44 ± 7 yrs (48 hrs), 48 ± 3 yrs (72 hrs), 48 ± 8 yrs (6 d) and 48 ± 6 yrs (10 d). The ten fastest men in 6 hrs and 12 hrs were younger than the ten fastest men in 72 hrs, 6 d and 10 d, respectively. Running speed was highest in 6 hrs (Figure 3) and decreased with increasing race duration (Table 1) in both women and men.

**Table 1:** Results of the ANOVA for differences in performance and age of peak performance between race durations

**Comparisons**	**Age**		**Running speed**	
	**Women**	**Men**	**Women**	**Men**
6 hrs *versus* 12 hrs	ns	ns	****	****
6 hrs *versus* 24 hrs	ns	ns	****	****
6 hrs *versus* 48 hrs	ns	ns	****	****
6 hrs *versus* 72 hrs	ns	**	****	****
6 hrs *versus* 144 hrs	ns	**	****	****
6 hrs *versus* 240 hrs	ns	**	****	****
12 hrs *versus* 24 hrs	ns	ns	****	****
12 hrs *versus* 48 hrs	ns	ns	****	****
12 hrs *versus* 72 hrs	ns	*	****	****
12 hrs *versus* 144 hrs	ns	*	****	****
12 hrs *versus* 240 hrs	ns	*	****	****
24 hrs *versus* 48 hrs	ns	ns	****	****
24 hrs *versus* 72 hrs	ns	ns	****	****
24 hrs *versus* 144 hrs	ns	ns	****	****
24 hrs *versus* 240 hrs	ns	ns	****	****
48 hrs *versus* 72 hrs	ns	ns	****	****
48 hrs *versus* 144 hrs	ns	ns	****	****
48 hrs *versus* 240 hrs	*	ns	****	****
72 hrs *versus* 144 hrs	ns	ns	ns	**
72 hrs *versus* 240 hrs	ns	ns	****	****
144 hrs *versus* 240 hrs	ns	ns	****	****

ns = non-significant, * = *p* < 0.05, ** = *p* < 0.01, **** = *p* < 0.0001.

### Age of the annual fastest

**Figure 4:**
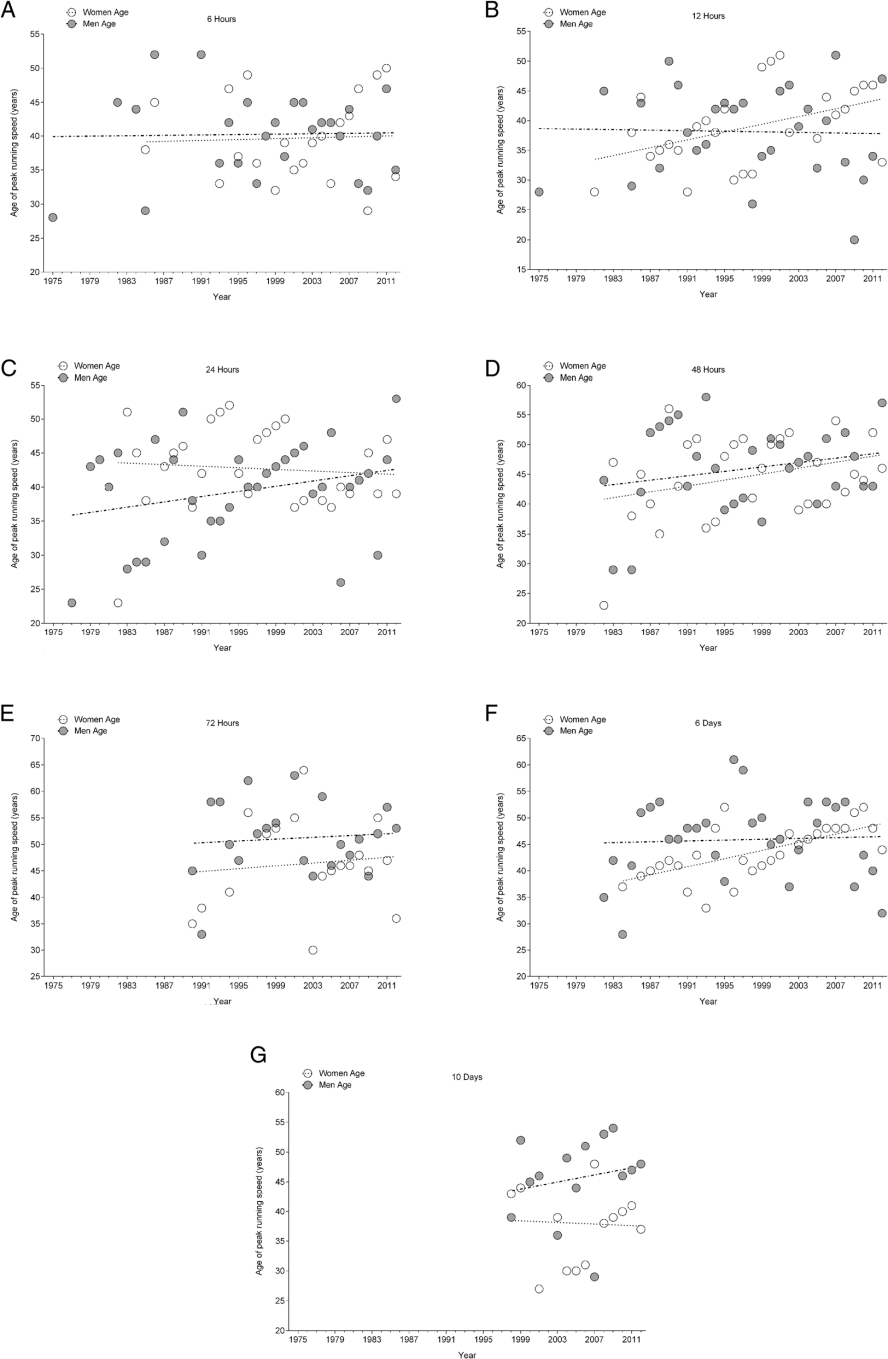
Changes in the age of the annual fastest women and men across the years in 6 hrs (Panel A), 12 hrs (Panel B), 24 hrs (Panel C), 48 hrs (Panel D), 72 hrs (Panel E), 6 d (Panel F) and 10 d (Panel G).

**Figure 5:**
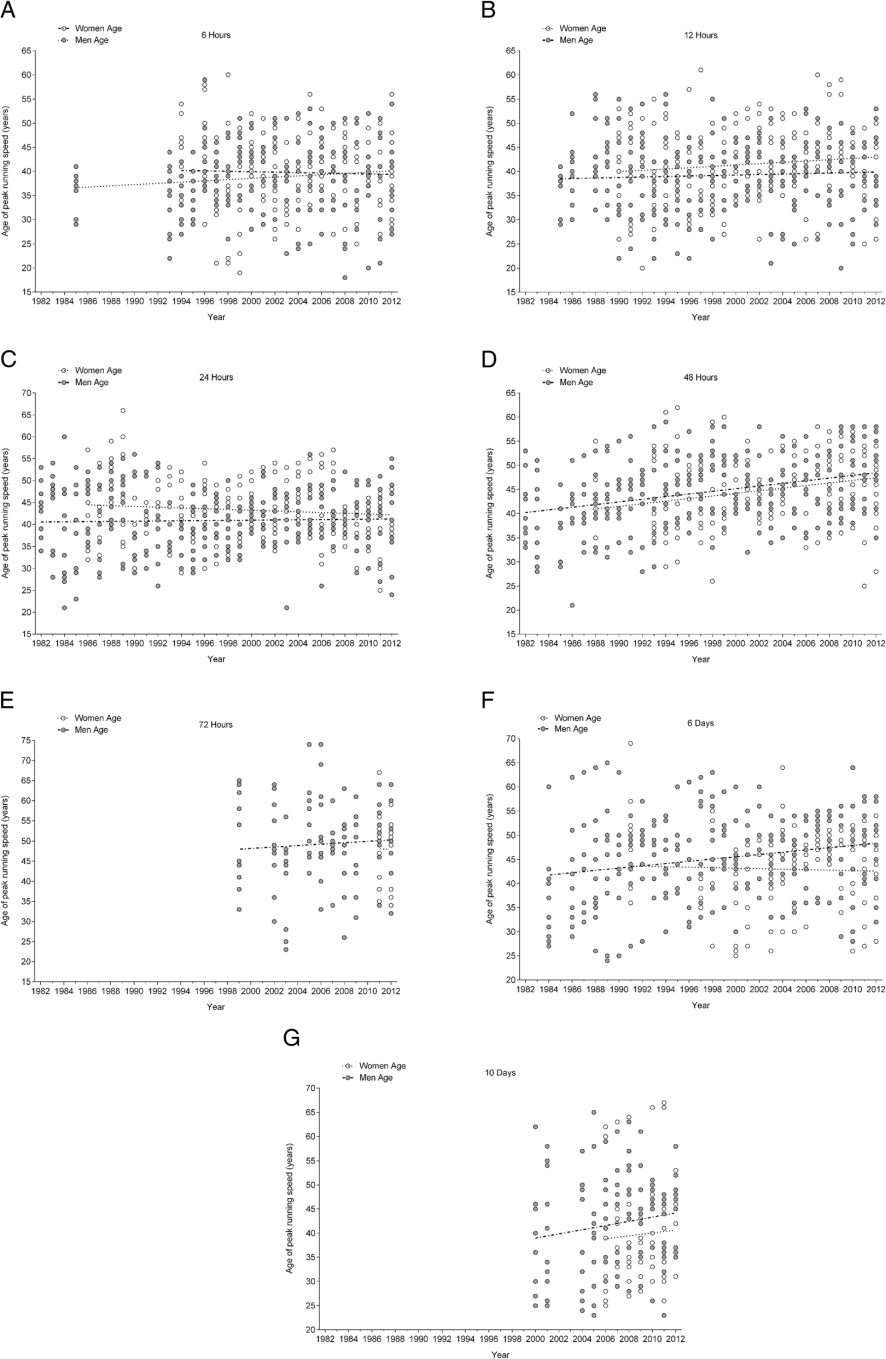
Changes in the age of the annual ten fastest women and men across the years in 6 hrs (Panel A), 12 hrs (Panel B), 24 hrs (Panel C), 48 hrs (Panel D), 72 hrs (Panel E), 6 d (Panel F) and 10 d (Panel G).

The athlete’s age of the annual fastest men (Figure 4) remained unchanged across years of competitions for all events from 6 hrs to 10 d (Table 2). For women (Figure 4), the athlete’s age of the fastest finishers increased in 12 hrs and 6 d (Table 3) across years of competitions. For the annual ten fastest women and men (Figure 5), the athlete’s age of the ten fastest finishers remained unchanged in 6 hrs, 12 hrs, 24 hrs, 72 hrs, and 10 days (Table 4). In 48 hrs, the athlete’s age of the ten fastest finishers increased for both women and men across years of competitions. In 6 d, the athlete’s age of the ten fastest women remained unchanged whereas it increased in men (Table 5).

**Table 2:** Multi-level regression analyses for the change in age across years for the annual fastest male and female runners with correction for multiple finishes

	***ß***	**SE (*ß*)**	**Stand. *ß***	**T**	***p***
**Annual fastest men**					
**6 hrs**	0.015	0.130	0.023	0.114	0.910
**12 hrs**	-0.023	0.155	-0.029	-0.151	0.881
**24 hrs**	0.193	0.118	0.275	1.642	0.110
**48 hrs**	0.184	0.147	0.229	1.248	0.223
**72 hrs**	0.084	0.222	0.084	0.377	0.710
**6 d**	0.039	0.154	0.047	0.252	0.803
**10 d**	0.296	0.432	0.194	0.686	0.506
**Annual fastest women**					
**6 hrs**	0.033	0.182	0.041	0.183	0.856
**12 hrs**	0.330	0.126	0.450	2.617	0.014*
**24 hrs**	-0.058	0.124	-0.086	-0.465	0.645
**48 hrs**	0.249	0.140	0.318	1.775	0.087
**72 hrs**	0.136	0.316	0.107	0.430	0.673
**6 d**	0.385	0.083	0.666	4.640	<0.0001*
**10 d**	-0.067	0.401	-0.048	-0.166	0.871

* = significantly different.

**Table 3:** Age of the annual fastest finishers in races held between 6 hrs and 10 d

**Race**	**Sex**	**Age of the runners at the start of the investigated period (yrs)**	**Age of the runners with no change across years (yrs)**	**Age of the runners at the end of the investigated period (yrs)**
**6 hrs**	Women		40 ± 6	
	Men		40 ± 6	
**12 hrs**	Women	28 (1975)		33 (2012)
	Men		38 ± 7	
**24 hrs**	Women		43 ± 6	
	Men		39 ± 7	
**48 hrs**	Women		45 ± 7	
	Men		46 ± 7	
**72 hrs**	Women		46 ± 8	
	Men		51 ± 7	
**6 d**	Women	37 (1984)		44 (2012)
	Men		46 ± 7	
**10 d**	Women		38 ± 6	
	Men		46 ± 7	

Results are presented as mean ± SD.

**Table 4:** Multi-level regression analyses for the change in age across years for the annual ten fastest male and female runners after correction for multiple finishes

	***ß***	**SE (*ß*)**	**Stand. *ß***	**T**	***p***
**Annual ten fastest men**					
**6 hrs**	0.129	0.076	0.117	1.693	0.092
**12 hrs**	0.052	0.056	0.056	0.925	0.356
**24 hrs**	0.023	0.045	0.030	0.518	0.605
**48 hrs**	0.275	0.043	0.347	6.390	<0.0001 *
**72 hrs**	0.177	0.277	0.064	0.638	0.525
**6 d**	0.236	0.062	0.224	3.833	<0.0001 *
**10 d**	0.435	0.262	0.158	1.658	0.100 *
**Annual ten fastest women**					
**6 hrs**	-0.045	0.114	-0.029	-0.392	0.696
**12 hrs**	0.138	0.074	0.123	1.865	0.063
**24 hrs**	-0.087	0.052	-0.103	-1.693	0.092
**48 hrs**	0.269	0.075	0.241	3.576	<0.0001 *
**72 hrs**	-0.200	4.419	-0.011	-0.045	0.964
**6 d**	-0.052	0.114	-0.036	-0.454	0.650
**10 d**	0.304	0.651	0.056	0.466	0.642

* = significantly different.

**Table 5:** Age of the annual ten fastest finishers in races held between 6 hrs and 10 d

**Race**	**Sex**	**Age of the runners at the start of the investigated period (yrs)**	**Age of the runners with no change across years (yrs)**	**Age of the runners at the end of the investigated period (yrs)**
**6 hrs**	Women		39.8 ± 7.7	
	Men		38.8 ± 7.1	
**12 hrs**	Women		41.5 ± 7.5	
	Men		39.2 ± 7.0	
**24 hrs**	Women		43.3 ± 6.2	
	Men		40.9 ± 6.6	
**48 hrs**	Women	41.2 ± 6.6 (1988)		43.8 ± 7.5 (2012)
	Men	40.3 ± 7.0 (1982)		50.2 ± 7.6 (2012)
**72 hrs**	Women		48.0 ± 9.9	
	Men		49.3 ± 10.1	
**6 d**	Women		42.9 ± 6.9	
	Men	36.9 ± 9.9 (1984)		47.7 ± 9.4 (2012)
**10 d**	Women		39.7 ± 10.5	
	Men		41.8 ± 10.2	

Results are presented as mean ± SD.

### Running speed of the annual fastest

**Figure 6:**
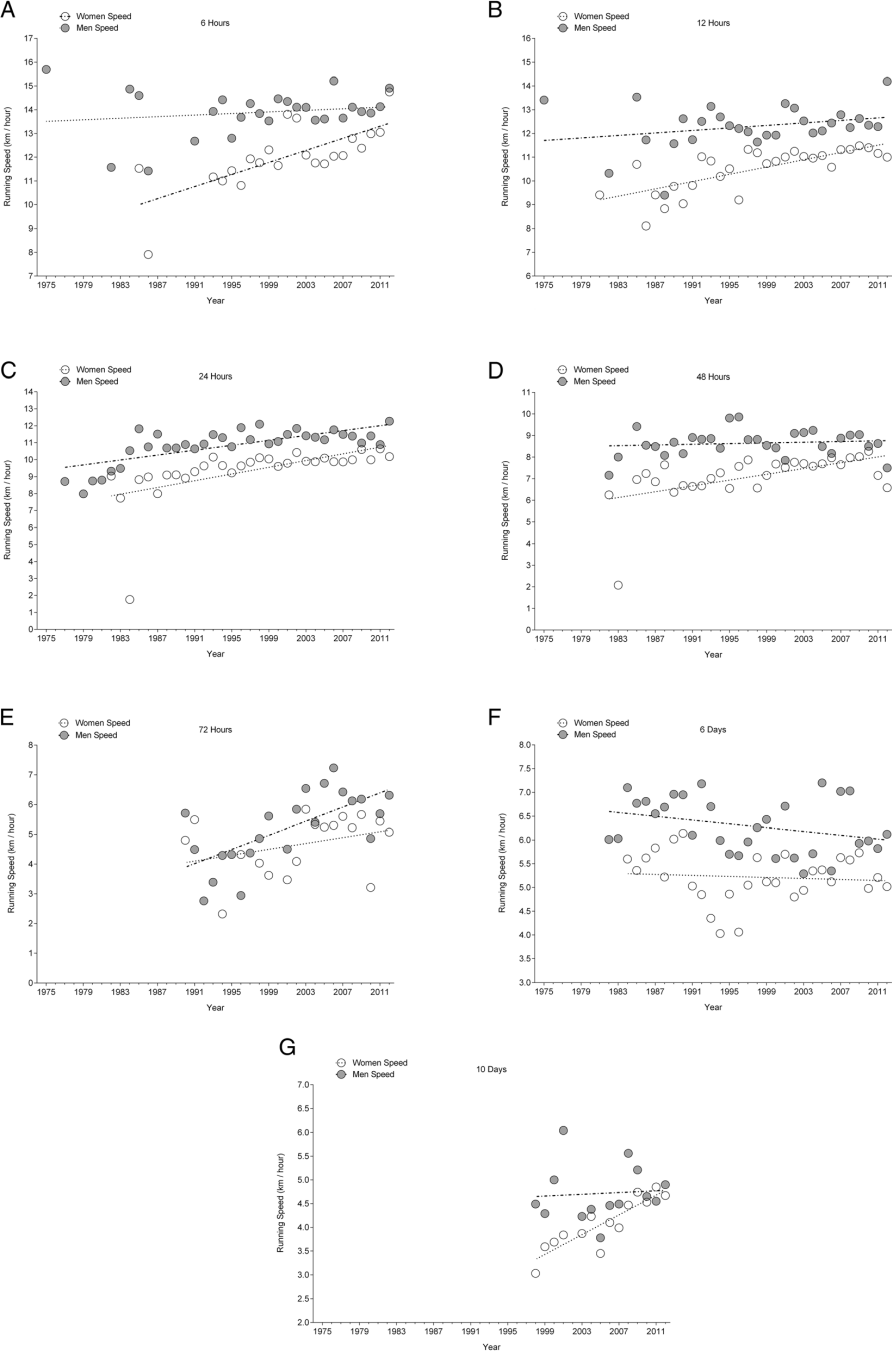
Changes in running speeds of the annual fastest women and men across the years in 6 hrs (Panel A), 12 hrs (Panel B), 24 hrs (Panel C), 48 hrs (Panel D), 72 hrs (Panel E), 6 d (Panel F) and 10 d (Panel G).

**Figure 7:**
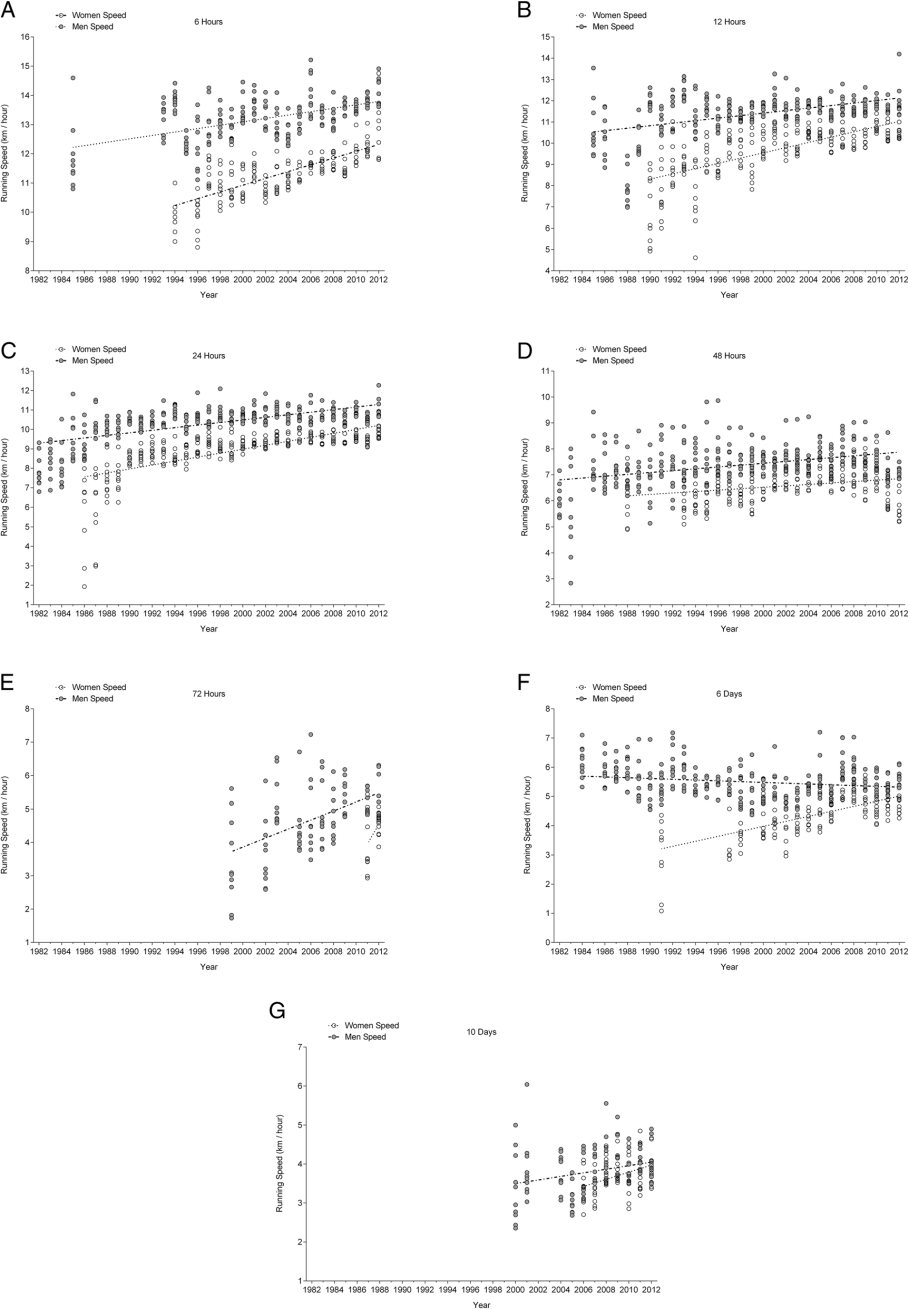
Changes in running speeds of the annual ten fastest women and men across the years in 6 hrs (Panel A), 12 hrs (Panel B), 24 hrs (Panel C), 48 hrs (Panel D), 72 hrs (Panel E), 6 d (Panel F) and 10 d (Panel G).

The annual fastest men improved running speed in 24 hrs and 72 hrs (Figure 6) across years of competitions, and the annual fastest women in all race durations except 72 hrs and 6 d (Table 6 and 7). The annual ten fastest women and men (Figure 7) improved running speed in race durations (Table 8) with the exception of 6 d in men where performance decreased (Table 9). Men improved running speed in 6 hrs (19.5%), 12 hrs (11.8%), 24 hrs (40.8%), 48 hrs (17.8%), 72 hrs (54.0%), and 10 d (16.6%). In 6 d, performance decreased by 8.4%. Women improved running speed in 6 hrs (28.1%), 12 hrs (50.4%), 24 hrs (57.5%), 48 hrs (13.1%), 72 hrs (14.4%), 6 d (45.6%), and 10 d (15.1%).

**Table 6:** Multi-level regression analyses for change in running speed across years for the annual fastest male and female runners after correction for multiple finishes and age of athletes with multiple finishes

	***ß***	**SE (*ß*)**	**Stand. *ß***	**T**	***p***
**Annual fastest men**					
**6 hrs**	0.018	0.016	0.183	1.095	0.285
**12 hrs**	0.027	0.018	0.272	1.445	0.161
**24 hrs**	0.075	0.013	0.747	5.891	<0.0001 *
**48 hrs**	0.013	0.012	0.193	1.036	0.310
**72 hrs**	0.126	0.023	0.712	5.443	<0.0001 *
**6 d**	-0.020	0.011	-0.316	-1.767	0.088
**10 d**	0.001	0.037	0.009	0.030	0.977
**Annual fastest women**					
**6 hrs**	0.129	0.026	0.730	4.959	<0.0001 *
**12 hrs**	0.075	0.015	0.722	4.866	<0.0001 *
**24 hrs**	0.099	0.027	0.578	3.739	0.001 *
**48 hrs**	0.074	0.021	0.604	3.628	0.001 *
**72 hrs**	0.058	0.031	0.386	1.872	0.081
**6 d**	0-.013	0.016	-0.212	-0.818	0.421
**10 d**	0.104	0.017	0.883	6.248	<0.0001 *

* = significantly different.

**Table 7:** Running speed of the annual fastest finishers in races held between 6 hrs and 10 d

**Race**	**Sex**	**Running speed of the runners at the start of the investigated period (km/h)**	**Running speed of the runners with no change across years (km/h)**	**Running speed of the runners at the end of the investigated period (km/h)**
**6 hrs**	Women	11.53 (1985)		14.75 (2012)
	Men		13.89 ± 0.96	
**12 hrs**	Women	9.41 (1981)		11.0 (2012)
	Men		12.30 ± 0.91	
**24 hrs**	Women	9.03 (1982)		10.18 (2012)
	Men	8.71 (1977)		12.27 (2012)
**48 hrs**	Women	6.25 (1982)		6.58 (2012)
	Men		8.64 ± 0.59	
**72 hrs**	Women		4.66 ± 1.00	
	Men	5.71 (1990)		6.31 (2012)
**6 d**	Women		5.21 ± 0.51	
	Men		6.29 ± 0.57	
**10 d**	Women	3.03 (1998)		4.76 (2012)
	Men		4.71 ± 0.58	

Results are presented as mean ± SD.

**Table 8:** Multi-level regression analyses for the change in running speed across years for the annual ten fastest male and female runners after correction for multiple finishes and age of athletes with multiple finishes

	***ß***	**SE (*ß*)**	**Stand. *ß***	**T**	***p***
**Annual ten fastest men**					
**6 hrs**	0.060	0.007	0.534	9.034	<0.0001 *
**12 hrs**	0.061	0.007	0.472	8.840	<0.0001 *
**24 hrs**	0.067	0.005	0.633	14.344	<0.0001 *
**48 hrs**	0.034	0.006	0.344	5.970	<0.0001 *
**72 hrs**	0.136	0.024	0.503	5.721	<0.0001 *
**6 d**	-0.014	0.004	-0.199	-3.295	0.001 *
**10 d**	0.044	0.016	0.260	2.773	0.007 *
**Annual ten fastest women**					
**6 hrs**	0.116	0.009	0.700	13.087	<0.0001 *
**12 hrs**	0.125	0.010	0.658	13.013	<0.0001 *
**24 hrs**	0.103	0.007	0.685	15.225	<0.0001 *
**48 hrs**	0.023	0.007	0.215	3.148	0.002 *
**72 hrs**	0.565	0.259	0.397	2.183	0.043 *
**6 d**	0.087	0.009	0.620	10.117	<0.0001 *
**10 d**	0.095	0.028	0.381	3.445	0.001 *

* = significantly different.

**Table 9:** Running speed of the annual ten fastest finishers in races held between 6 hours and 10 days

**Race**	**Sex**	**Running speed of the runners at the start of the investigated period (km/h)**	**Running speed of the runners at the end of the investigated period (km/h)**
**6 hrs**	Women	9.93 ± 0.54 (1994)	12.72 ± 0.98 (2012)
	Men	11.80 ± 1.16 (1985)	14.10 ± 0.47 (2012)
**12 hrs**	Women	6.94 ± 1.60 (1990)	10.44 ± 0.25 (2012)
	Men	10.71 ± 1.30 (1985)	11.98 ± 0.84 (2012)
**24 hrs**	Women	6.23 ± 2.34 (1986)	9.81 ± 0.25 (2012)
	Men	7.86 ± 0.81 (1986)	11.07 ± 0.51 (2012)
**48 hrs**	Women	6.27 ± 0.96 (1988)	7.09 ± 0.60 (2012)
	Men	6.02 ± 0.58 (1982)	7.09 ± 0.20 (2012)
**72 hrs**	Women	3.96 ± 0.91 (2011)	4.53 ± 0.34 (2012)
	Men	3.46 ± 1.33 (1999)	5.33 ± 0.64 (2012)
**6 d**	Women	3.22 ± 1.28 (1991)	4.69 ± 0.27 (2012)
	Men	6.18 ± 0.53 (1984)	5.66 ± 0.28 (2012)
**10 d**	Women	3.45 ± 0.43 (2006)	3.97 ± 0.43 (2012)
	Men	3.38 ± 0.92 (2000)	3.94 ± 0.51 (2012)

Results are presented as mean ± SD.

## Discussion

This study intended to determine the athlete’s age of peak running speed in ultra-marathoners competing in time-limited race held for 6 hrs, 12 hrs, 24 hrs, 48 hrs, 6 d and 10 d between 1975 and 2012 with the hypothesis that the athlete’s age of peak ultra-marathon performance would increase with the duration of the event. In contrast to the hypothesis, the athlete’s age of peak ultra-marathon performance did not increase with rising race duration.

### Differences in the athlete’s age of peak performance between women and men

Regarding the ten fastest women and men ever, the athlete’s age of the ten fastest women was different between 48 hrs and 240 hrs, but no differences were found between the other race durations. For men, the ten fastest men in 6 hrs and 12 hrs were younger than the ten fastest men in 72 hrs, 6 d and 10 d, respectively. Potential explanations for these sex differences could be (*i*) differences in participations trends between women and men, (*ii*) differences between women and man in motivation to compete, and (*iii*) differences in previous experience between women and men in ultra-marathon running.

Although participation increased in both women and men for all event durations in an exponential manner, female participation was considerably lower compared to men’s participation. Generally, women account for ~10-20% in ultra-running competitions [2,3] although female participation increased in recent years in ultra-marathon running across years of competitions [15,16].

Different motivational factors between women and men might also be a potential reason to explain the differences in the athlete’s ages [17-19]. For female ultra-marathoners, task orientation (*i.e.* finishing an ultra-marathon or accomplishing various goals) was more important than ego orientation (*i.e.* placing in the top three overall or beating the concurrent) [18]. Generally, men are over-represented in sports and distance running is an ideal domain for male predisposition for enduring competitiveness or long-term motivation to improve one's performance [19]. Distance running indicates enduring competitiveness, allows objective comparisons, and is accessible, acceptable, and popular for both men and women [19]. More men than women run relatively fast in the United States of America which is attributed to men's greater training motivation [19].

The aspect of experience might be important to explain the dominance of master runners in ultra-distances [20,21]. In 161-km ultra-marathons held in North America between 1977 and 2008, the number of annually completed races by an individual athlete increased across years [22]. Successful ultra-marathoners generally train for ~7 yrs before competing in the first ultra-marathon [21] and have ~7 yrs of experience in ultra-marathon running [20]. Active ultra-marathoners train for ~3,300 km per year where the annual running distance is related to age and the longest ultra-marathon held within that year [23]. Hoffman and Parise [21] showed for 161-km ultra-marathoners that high-level performances can be sustained late into the fourth decade of life but subsequent aging is associated with declines in performance and the adverse effects of aging on performance can be offset by greater experience in these events.

It is a common finding that successful ultra-marathoners are older than 35 yrs [2,8,20,23-25]. Wegelin and Hoffman [26] showed that especially women performed better than men above the age of 38 yrs. Additionally, ultra-marathoners seemed to be well-educated middle-aged men since most of the successful 161-km ultra-marathoners have a high education. Hoffman and Fogard [25] reported that 43.6% of 161-km ultra-marathoners had a bachelor degree and 37.2% and graduate degree.

### Ultra-marathoners improved their running performance

Another important finding was that the fastest finishers improved running speed across years of competition in all races although they showed no change in the athlete’s age of peak performance. Similar findings have been reported for the analysis of 161-km ultra-marathoners competing worldwide where performance improved and the athlete’s age of peak performance remained unchanged across years of competition [3]. The annual top ten performances improved in 161-km ultra-marathoners by 13.7% from 1998 to 2011 for women and by 14.5% for men [3]. The mean athlete’s ages of the annual top ten fastest runners were ~39 yrs for women and ~37 yrs for men [3]. The athlete’s age of peak running performance was not different between women and men and showed no changes across years of competition [3].

The present findings differ, however, from results reported by Hoffman *et al.*[22] investigating performance trends in 161-km ultra-marathons held in North America between 1977 and 2008. The average race times of the fastest runners showed no change across years of competition for any age group for either sex. The different findings between the present study and the study of Hoffman *et al.*[22] might be explained by the different samples. While Hoffman *et al.*[22] investigated 161-km ultra-marathons held within one continent (North America) we investigated the total of all time-limited races held worldwide.

This finding for ultra-marathoners is in contrast to recent findings in triathletes competing in ‘Ironman Hawaii’ where the athlete’s age in the annual top ten finishers increased although the athletes became faster 1983 and 2012 [27]. However, this trend could be confirmed in long-distance triathletes competing in ‘Ultraman Hawaii’ where the annual top three women and men improved their performance during the 1983–2012 period although the athlete’s age of the annual top three women and men increased [28].

Similar findings of an increase of the athlete’s age of peak athletic performance with rising race distance have been reported for swimmers [29-31] and triathletes [32-34]. In elite pool swimmers, men (~24 yrs) achieved peak performance later than women (~22 yrs) [29]. Similar athlete’s ages of peak performance were reported for long-distance open-water swimmers [31]. In the 5 km, 10 km and 25 km FINA (Fédération Internationale de Natation) World Cup swimming events held from 2000 to 2012, the age of peak swimming speed remained stable at ~22 yrs in 5 km, at ~23 yrs in 10 km and at ~23 yrs in 25 km for the annual top ten women [31]. For the annual top ten men, the athlete’s age of peak swimming speed increased from ~24 to ~28 yrs in 10 km but remained stable at ~25 yrs in 5 km and at ~27 yrs in 25 km [31]. Older athlete’s ages, however, were reported for open-water ultra-distance swimmers in longer race distances [30]. At the 46-km 'Manhattan Island Marathon Swim', the athlete’s age of the annual three fastest swimmers increased from ~28 yrs (1983) to ~38 yrs (2013) in women and from ~23 yrs (1984) to ~42 yrs (2013) in men [30]. For Olympic distance triathletes (*i.e.* 1.5 km swimming, 40 km cycling and 10 km running), the athlete’s ages of peak performance were similar (~28 yrs) for men and women [33]. In long-distance triathlon, however, it seemed that the athlete’s age of peak performance increased with rising race distance [32,34]. For the Ironman distance (*i.e.* 3.8 km swimming, 180 km cycling and 42 km running), the athlete’s age of peak Ironman triathlon performance was ~32 yrs for men and ~33 yrs for women [34]. For the Triple Iron ultra-triathlon distance (*i.e.* 11.4 km swimming, 540 km cycling and 126.6 km running), the mean athlete’s age of the fastest male finishers was ~38 yrs and increased to ~41 yrs for athletes competing in a Deca Iron ultra-triathlon (*i.e.* 38 km swimming, 1,800 km cycling and 422 km running) [32].

### Limitations

The results may not apply to distance limited races, such as 50-mile and especially 100-mile ultra-marathons that are often on trails with significantly more elevation gain and loss than time-limited event. The top finishers in difficult 100-mile trail ultramarathons are now frequently in their 20’s [35-37].

## Conclusion

The present findings show that for the fastest women ever in time-limited race between 1975 and 2012, the lowest age was in 10 d (~37 yrs) and highest in 48 hrs (~46 yrs). For men, the athlete’s age of the ten fastest in 6 hrs (~35 yrs) and 12 hrs (~37 yrs) was lower than the athlete’s age of the ten fastest in 72 hrs (~48 yrs), 6 d (~48 yrs) and 10 d (~48 yrs). The assumption that the age of peak ultra-marathon performance increased with increasing race distance could not be confirmed. Although these athletes were master runners by definition, they improved their performance across years of competition. The differences in the athlete’s age of peak performance between the sexes for the different race durations need further investigations. Future studies are required to understand the motivation of master ultra-runners and to examine their specific training, physiology and anthropometric characteristics.

## Competing interests

The authors declare that they have no competing interests.

## Authors’ contributions

CR performed the statistical analyses and drafted the manuscript, BK and MZ collected the data and helped in drafting the manuscript, TR helped in drafting the manuscript. All authors read and approved the final manuscript.

## Pre-publication history

The pre-publication history for this paper can be accessed here:

http://www.biomedcentral.com/2052-1847/6/36/prepub
